# Effect of Bile Pigments on the Compromised Gut Barrier Function in a Rat Model of Bile Duct Ligation

**DOI:** 10.1371/journal.pone.0098905

**Published:** 2014-06-03

**Authors:** Kangkang Zhou, Mingshan Jiang, Yuanli Liu, Yilin Qu, Guojing Shi, Xinguang Yang, Xiaofa Qin, Xiuhong Wang

**Affiliations:** 1 Department of Biochemistry and Molecular Biology, Heilongjiang Provincial Science and Technology Innovation Team in Higher Education Institutes for Infection and Immunity, Harbin Medical University, Harbin, China; 2 Department of General Surgery, The Second Affiliated Hospital of Harbin Medical University, Harbin, China; 3 Department of Surgery, Rutgers-New Jersey Medical School, Newark, New Jersey, United States of America; Emory University School of Medicine, United States of America

## Abstract

**Background:**

Studies have shown that the absence of bile in the gut lumen, either by bile duct ligation or bile diversion, induces mucosal injury. However, the mechanism remains elusive. In this study, the role of bile pigments in gut barrier function was investigated in a rat model of bile duct ligation.

**Methods:**

Male Sprague Dawley (SD) rats were used in this study. After ligation of bile duct, the animals were administrated with free bilirubin, bilirubin ditaurate, or biliverdin by intragastric gavage. 1, 2, or 3 days later, the animals were sacrificed and the damage of mucosa was assessed by histological staining as well as biochemical parameters such as changes of diamine oxidase (DAO) and D-lactate (D-Lac) in the blood. Trypsin and chymotrypsin of the gut were also measured to determine how these digestive proteases may relate to the observed effects of bile pigments.

**Results:**

Bile duct ligation (BDL) caused significant increases in gut trypsin and chymotrypsin along with damage of the mucosa as demonstrated by the histological findings under microscope, the reduced expression of tight junction molecules like occludin, and significant changes in DAO and D-lac in the blood. Free bilirubin but not bilirubin ditaurate or biliverdin showed significant inhibitions on trypsin and chymotrypsin as well as alleviated changes of histological and biochemical parameters related to gut barrier disruption.

**Conclusion:**

Bile may protect the gut from damage through inhibiting digestive proteases like trypsin and chymotrypsin by free bilirubin.

## Introduction

Multiple studies showed that the absence of bile in the gut, as seen in animals with either bile duct ligation or bile diversion, leads to mucosal injury, which was demonstrated by morphological changes such as villous atrophy, villous edema, and lacteal canal dilatation, increased intestinal permeability, and increased translocation of bacteria from the gut to other organs like the mesenteric lymph nodes, liver, and spleen [1]–[5]. This suggests some components in the bile may have played a critical role in maintaining gut barrier function. It has been well documented that bile acids, one of the main components in the bile, are harmful to the mucosa of the gastrointestinal tract [6]–[9]. The beneficial effect of bile on gut barrier remains to be determined.

Besides bile salt, digestive protease like trypsin has been found another damaging factor for mucosa. For instance, both bile salt and trypsin exacerbated the damage of mucosa by radiation or alkali [10], [11]. As studies revealed that free bilirubin but not conjugated bilirubin or biliverdin may inhibit the activity of digestive proteases like trypsin and chymotrypsin [12], [13], this may provide an explanation as to how the gut is protected against the damage by digestive proteases.

In humans and some other animals, bilirubin is excreted as the catabolite of haem. Bilirubin in the bile is mainly in the conjugated form (bilirubin glucuronides) [14], thus the digestion of the dietary proteins will not be affected. Conjugated bilirubin can be hydrolyzed to unconjugated bilirubin by β-glucuronidase [15], which exists in both eukaryotic cells and bacteria [16]. The unconjugated bilirubin layer thus formed may exert an effective protection of the gut against the digestive damage. The metabolism of the conjugated biliary bilirubin and the inhibition of digestive proteases would be two sequential events. In this study, the role of different bile pigments in gut barrier function was investigated using a rat model of bile duct ligation.

## Materials and Methods

### Antibodies, chemicals, reagents and other materials

Rabbit anti-occludin polyclonal IgG was acquired from Santa Cruz Biotechnology (CA, USA). Rabbit anti-glyceraldehyde 3-phosphate dehydrogenase (GAPDH) polyclonal IgG was purchased from XianZhi Biotechnology (Hangzhou, China). Rabbit anti-β-actin polyclonal IgG was acquired from Biosynthesis Biotechnology (Beijing, China), Horseradish peroxidase-labeled goat anti-rabbit IgG was obtained from ZSGB-Bio (Beijing, China).

Unconjugated bilirubin (UCB), biliverdin hydrochloride, peroxidase (POD), o-dianisidine dihydrochloride, cadaverine dihydrochloride, nicotinamide adenine dinucleotide (NAD), diamine oxidase (DAO) standard and D-Lactic dehydrogenase (D-Lac) standard were obtained from Sigma-Aldrich (Shanghai, China). Bilirubin ditaurate disodium was purchased from Frontier Scientific (Logan, Utah, USA). Dulbecco's phosphate buffered saline (PBS) and pentobarbital sodium were purchased from Solarbio (Beijing, China). All other reagents and solvents were purchased from J&k scientific (Beijing, China).

### Preparation of reagent

UCB was purified as described by McDonagh and Assisi [17]. The sodium salts of UCB and biliverdin were formed according to the method of Bulmer [18]. Then UCB and biliverdin were dissolved in PBS to the desired concentrations (0.1 to 10 mM), sonicated and filtered prior to administration. Solubilizing UCB and biliverdin in this manner resulted in the formation of optically clear solutions, without visible aggregation. Bilirubin ditaurate, supplied as a disodium salt, was used as is. UCB stock solutions (10 mM) were further diluted with PBS and administered intragastrically at various concentrations. All the experiments with bile pigments were performed under light protection to avoid photodegradation and autooxidation.

### The surgical procedure of BDL

Experimental bile diversion was achieved by catheterization of the common bile duct. The absence of bile in the intestine, as seen in animals with either bile duct ligation or bile diversion, promotes mucosal injury and bacterial translocation [3], [4], which would suggest that some components in the bile may have played a critical role in maintaining gut barrier function. The surgical procedure was performed under sterile conditions. Rats were anesthetized with pentobarbital sodium (50 mg/kg, intraperitoneally). Midline laparotomy was performed for identifying the bile duct. Under the dissecting microscope, the bile duct was then isolated, doubly ligated and transected between two ligatures. After BDL, rats were fed normally.

### Test systems used and experimental design

Adult male SD rats (220–330 g) were randomized so that each group has similar average body weight (270±20 g, n = 8), then the animals were fasted overnight with free access to water. For the BDL experiments, rats were anesthetized by intraperitoneal injection (IP) of pentobarbital sodium (50 mg/kg) and divided into control group (0 h), BDL 24, 48 and 72 h groups. After BDL, blood was collected from abdominal aorta with gel & clot activator tube (Kangjian, Taizhou, China). The blood samples were centrifuged (3000×g, 15 min) and the supernatant (plasma) was aliquotted, frozen on dry ice and later transferred to a −80°C freezer. The middle small intestine tissues were excised and either snap frozen at −80°C or stored in 10% formaldehyde solution until further use. For the intragastric administration of UCB experiments, rats were anesthetized and divided into 10 groups. After BDL, rats were administered intragastrically with 1 mL of UCB the following concentrations: 10 mM, 5 mM, 2.5 mM, 1.25 mM, 0.6 mM, 0.3 mM, 0.1 mM, 0.075 mM, 0.05 mM, 0.01 mM. For the intragastric administration of different bile pigments experiments, rats were anesthetized and divided into 3 groups. After BDL, rats were administered intragastrically with 1 mL of 0.1 mM UCB, bilirubin ditaurate and biliverdin, respectively. The blood samples and intestine tissues in these two groups were collected as described in the BDL experiment after 48 h.

### Ethics and Data Availability Statement

After collection of the blood and harvest of the tissues, the animals were euthanized by severing the aorta. Animal studies were approved by the regulations and protocol of the ethic committees of Harbin Medical University. All data are available upon request.

### Determination of trypsin and chymotrypsin concentrations

The concentration of trypsin in middle small intestine was assessed by rat trypsin ELISA kit (Xinqidi Biological Technology Co. LTD, Wuhan, China), according to the manufacturer's instructions. Results were obtained as ng/mL. The concentration of chymotrypsin was detected by rat chymotrypsin ELISA kit (Cusabio Biotech Co. LTD, Wuhan, China). Results were expressed as U/L.

### Measurement of DAO levels

The serum concentrations of DAO were assayed by spectrophotometric assay, 0.5 mL serum was incubated at 37°C for 30 min with the following compounds: 3 mL, 0.2 M PBS, 0.1 mL (4 µg) POD solution, 0.1 mL (500 µg) o-dianisidine dihydrochloride solution and 0.1 mL (175 µg) cadaverine dihydrochloride solution. After 30 min, DAO concentration in the serum was determined by direct measurement of the absorbance at 436 nm. Results were expressed as U of DAO per mL of serum.

### Measurement of D-Lac levels

Determination of D-Lac concentrations in serum was measured according to the method of Brandt [19]. Results were expressed as µg of D-Lac per mL of serum.

### Western blot analysis

The expression of occludin protein was determined by Western blot analysis. Briefly, 50 µg of total protein was extracted from intestinal mucosa using the RIPA lysis buffer (Beyotime, Shanghai, China) and protein concentration was quantitated by BCA method (Beyotime, Shanghai, China). The extracts were separated on a 10% sodium dodecyl sulphate-polyacrylamide gel electrophoresis (SDS-PAGE). Following electrophoretic transfer onto a nitrocellulose membrane and blocking with 5% milk solution, the blots were incubated with primary antibody overnight at 4°C [rabbit anti-occludin (1∶200), rabbit anti-GAPDH (1∶1000) or rabbit anti-β-actin (1∶800)] and with horseradish peroxidase-labelled secondary antibody [anti-rabbit (1∶5000)] for 2 h at room temperature. Protein bands were visualized by autoradiography with Hyperfilm ECL. GAPDH and β-actin were used as control protein, and the relative intensities of protein bands were analyzed by using the Quantity One (version 4.5.2) program (Bio-Rad Laboratories).

### Histological scoring of intestinal tissue

Intestine tissues from different experimental groups were fixed in 10% formaldehyde solution, paraffin embedded, sliced (5 µm in thickness) and then the slices were stained with hematoxylin and eosin (HE) staining and examined using a Olympus IX71 inverted fluorescence microscope (Olympus corporation, Tokyo, Japan). Histological scoring was assigned by blindly assessing the middle small intestine epithelial damage (goblet cell loss, crypt hyperplasia and ulcer size), inflammatory cell infiltration (mucosa, submucosa and muscle/serosa), resulting in a total scoring from 0 to 12, as Gruber described [20].

### Statistical Analysis

Results of at least three independent experiments were expressed as mean ± SD. Differences between groups were determined by one-way ANOVA with Dunnett's or Bonferroni's multiple comparisons post hoc tests, using InStat version 5.00 (GraphPad Software, San Diego, CA, USA). The correlations were assessed using linear or nonlinear regression in MS Excel (Microsoft corp, Redmond, WA, USA). Statistical significance was considered when P values were lower than 0.05.

## Results

### Effect of BDL on trypsin, chymotrypsin levels

We began our studies by evaluating the effect of BDL on trypsin and chymotrypsin levels, since the structural proteins of the human body may just be as vulnerable as the dietary proteins they digest.

**Figure 1:**
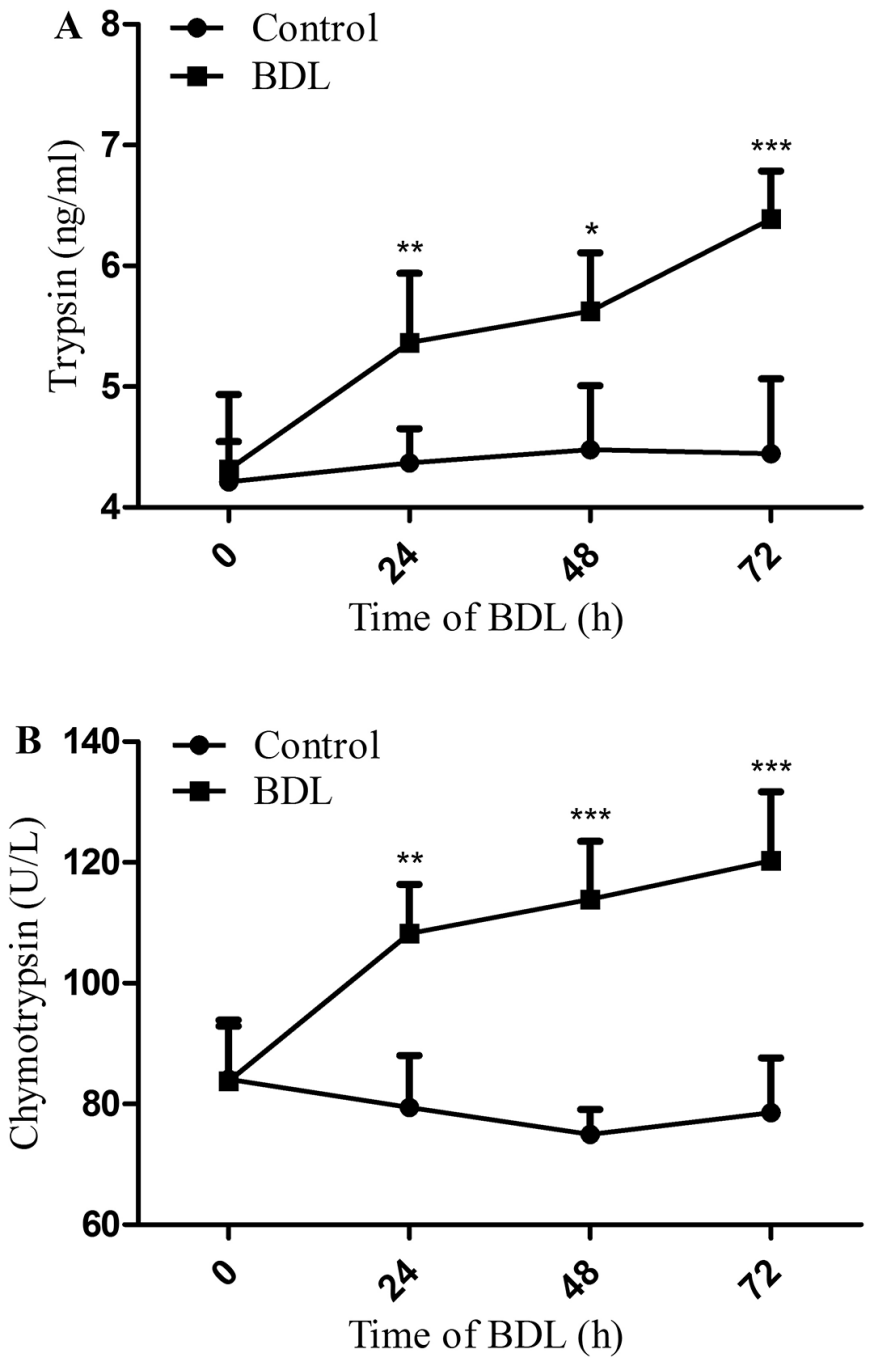
Effect of BDL on trypsin, chymotrypsin levels. After BDL, the amounts of trypsin (A) and chymotrypsin (B) in the middle small intestine were assayed by ELISA kit. Results are mean ± SD from at least three independent experiments. *p<0.05 **p<0.01 ***p<0.001 vs. respective control.

Adult male SD rats had their bile duct ligated for 24, 48 and 72 h. As shown in Figure 1, the amounts of trypsin and chymotrypsin in BDL groups increased in a time-dependent manner and significantly higher in respect to the controls (untreated rats) over the course of 72 h. The concentration of trypsin in BDL group was 1.23 folds higher (p<0.01) than in control group at 24 h and remained elevated thereafter (1.26 folds higher at 48 h, p<0.05 and 1.44 folds higher at 72 h, p<0.001). Similar to the trypsin, the chymotrypsin concentration in BDL group was 1.36 folds higher (p<0.01) in respect to the control at 24 h and elevated thereafter (1.52 folds higher at 48 h and 1.53 folds higher at 72 h respectively, p<0.001).

### Markers and the histopathologic changes of gut barrier disruption

**Figure 2:**
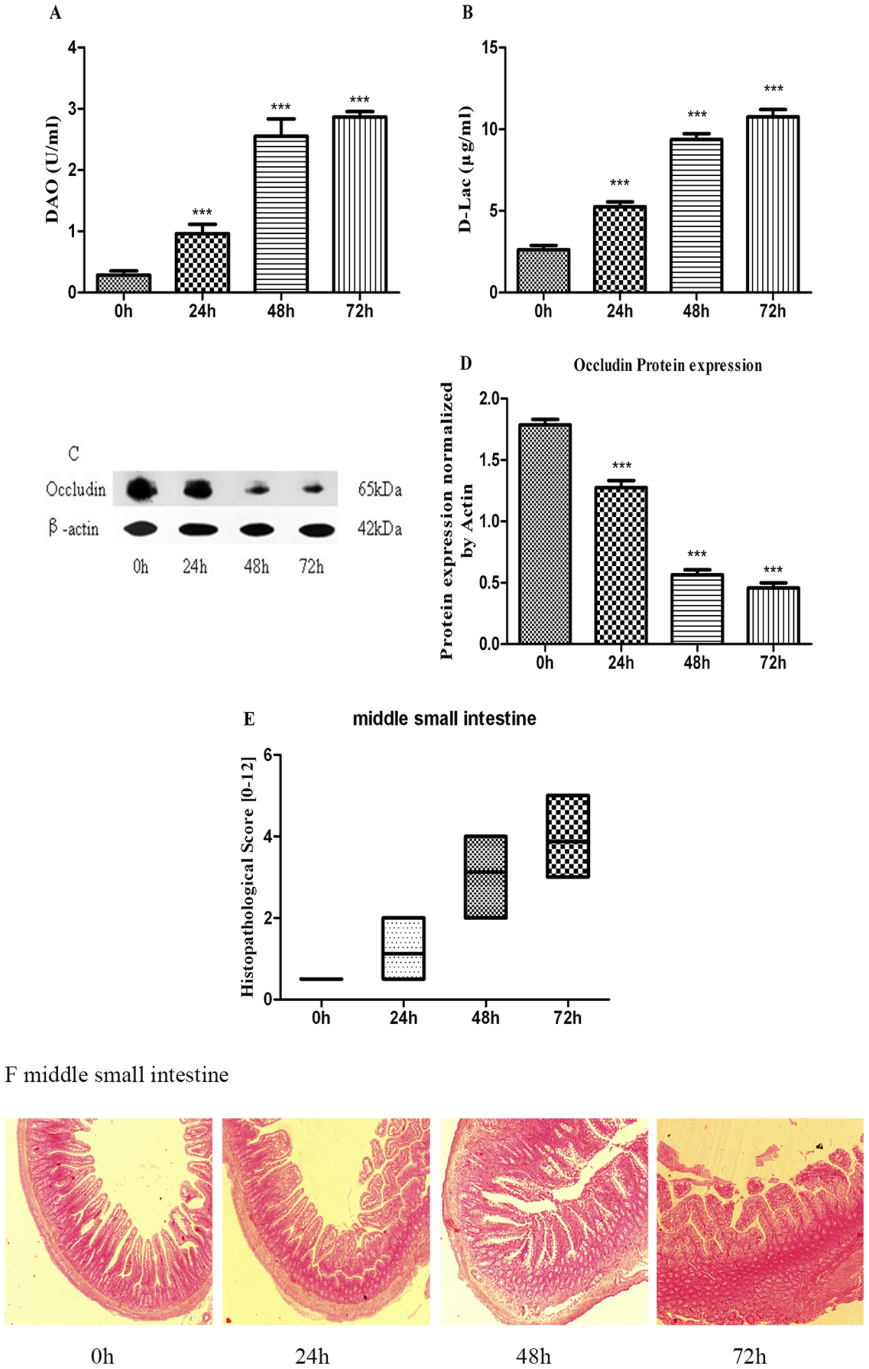
Markers and the histopathologic changes of gut barrier disruption. After BDL, the serum concentrations of DAO (A) and D-Lac (B) were assayed by spectrophotometric assay. Protein expression of occludin (65 kDa) was analyzed by Western blot (C). Occludin bands were normalized by actin and expressed as relative to control (D). Intestinal slices were stained with HE staining and analyzed by inverted fluorescence microscope, middle small intestine were blindly assessed for the degree of histopathology (E), all photos were captured at ×40 magnification (F). Results are mean ± SD from at least three independent experiments. ***p<0.001 vs. respective control.

Next, we evaluated the effects of BDL on DAO, D-Lac levels in serum as well as the barrier protein expression of occludin in epithelial tight junction of intestinal mucosa, as these proteins are important indicators of intestinal permeability and integrity [21]–[23]. The results are reported in Figure 2.

A time-dependent elevation in the serum concentrations of DAO and D-Lac were observed over the course of 72 h. As seen in Figure 2, the concentration of DAO in serum was observed with 3.4 folds higher (p<0.01) compared to the control at 24 h, and higher values were achieved (8.9 folds higher at 48 h and 10 folds higher at 72 h compared to the control, respectively, p<0.001). Additionally, BDL triggered a statistically significant elevation of D-Lac release (2 folds, 3.6 folds and 4.1 folds higher in respect to the control over the course of 72 h, respectively, p<0.001). On the contrary, the barrier protein expression of occludin was dramatically reduced under BDL (Figure 2C). Reduction of the expression was 28.7% (p<0.01) at time of 24 h, with further aggravation under BDL (68.4% at 48 h and 74.4% at 72 h compared to the control, respectively, p<0.001). These reflected a time-dependent loss of the permeability and integrity of intestinal mucosa.

We also studied the development of intestinal inflammation in BDL rats (Figure 2E and Figure 2F). In control group, the intestinal mucosa displayed structural-clarity and integrity. At 24 h of BDL, the middle small intestine tissue histopathology was more severe compared to the control, with further aggravation at 48 and 72 h, and the intestine tissue was significantly more severely inflamed at 72 h. The time of BDL for rats was determined as 48 h and this time was used in future experiments.

The concentrations of trypsin and chymotrypsin in middle small intestine were positively linearly correlated with DAO (r = 0.911, 0.896, respectively) and D-Lac (r = 0.91, 0.927, respectively) levels in serum (Table 1). In addition, trypsin and chymotrypsin levels were negatively linearly correlated with the expression of occludin (r = −0.966, −0.887, respectively). BDL caused significant increases in gut trypsin and chymotrypsin along with the gut barrier disruption.

**Table 1:** Linear-regression analysis of digestive proteases and the markers of gut barrier disruption over the course of 72 h.

Group	Pearson correlation (r)		
	DAO	D-Lac	Occludin
Trypsin	0.911	0.91	−0.966
Chymotrypsin	0.896	0.927	−0.887

### Determination of UCB concentration

The intestinal accumulation of digestive proteases contributed largely to gut injury, as digestive proteases could be inhibited by UCB in experimental model [12], we hypothesize that UCB possesses the same property in vivo. By measuring the serum concentrations of DAO and D-Lac we were able to evaluate the protective effect of UCB on mucosa.

**Figure 3:**
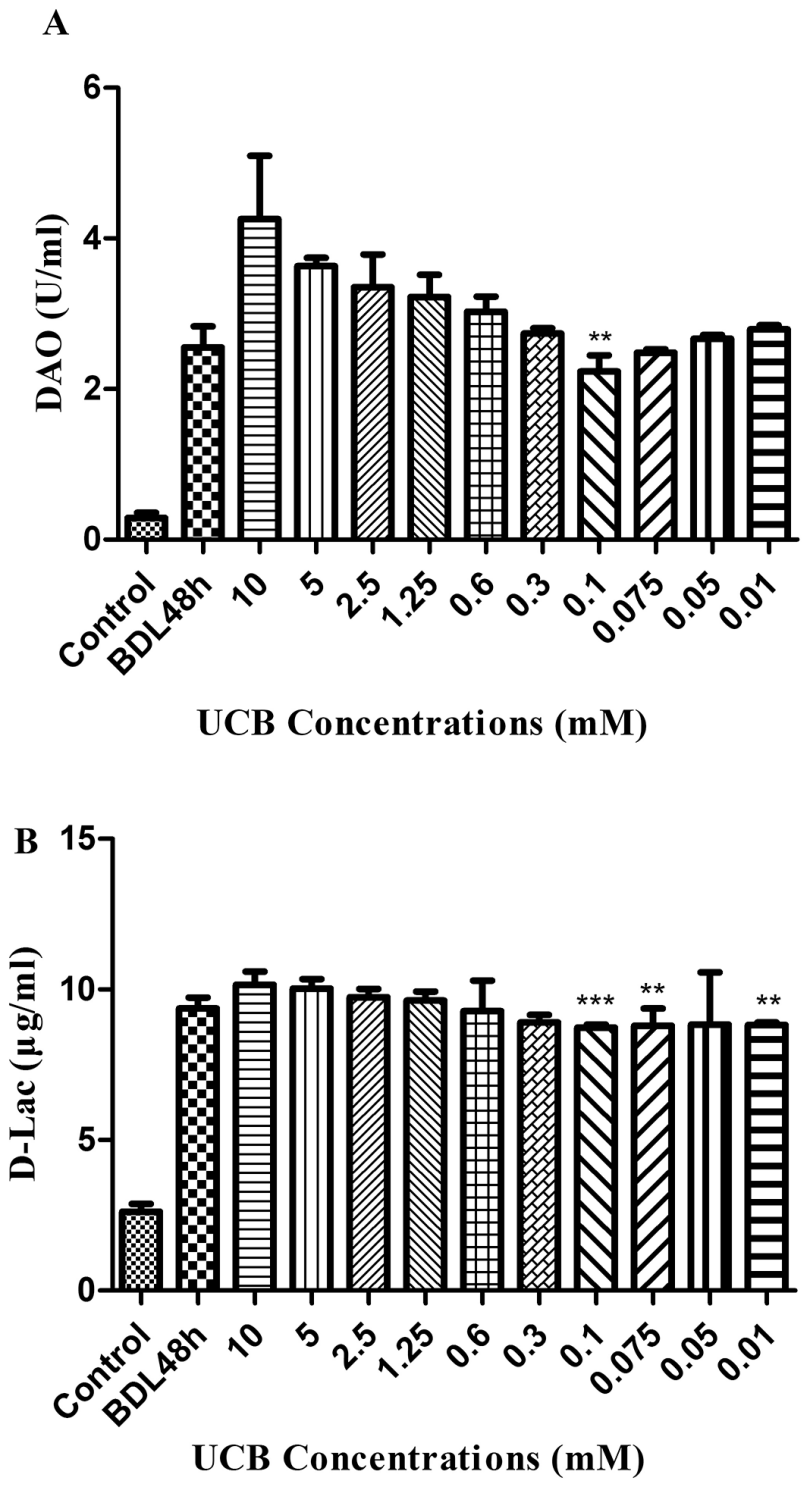
Determination of UCB concentration. After ligation of bile duct, the animals were administrated with free bilirubin at various concentrations by intragastric gavage. The serum concentrations of DAO (A) and D-Lac (B) were assayed by spectrophotometric assay after 48 h. Results are mean ± SD from at least three independent experiments. **p<0.01 and ***p<0.001 vs. respective BDL 48 h group.

UCB stock solutions (10 mM) were diluted with PBS and administered intragastrically at various concentrations. As shown in Figure 3, UCB triggered a mild, but statistically significant, inhibition of DAO and D-Lac release, which attained minimum levels of 2.23 U/ml and 8.73 µg/ml at 0.1 mM UCB administration group, respectively. The most effective concentration of UCB was determined as 0.1 mM, and this concentration was used in future experiments for examine the protective effect of UCB on gut.

### Effect of 0.1 mM bile pigments on trypsin, chymotrypsin levels

Some in vitro experiments revealed that free bilirubin but not conjugated bilirubin or biliverdin may inhibit the activity of digestive proteases [12], [13]. 0.1 mM UCB is assuned to be the most effective concentration in vivo, we began our studies by valuating the effects of 0.1 mM of different bile pigments on digestive proteases to demonstrate our speculation.

**Figure 4:**
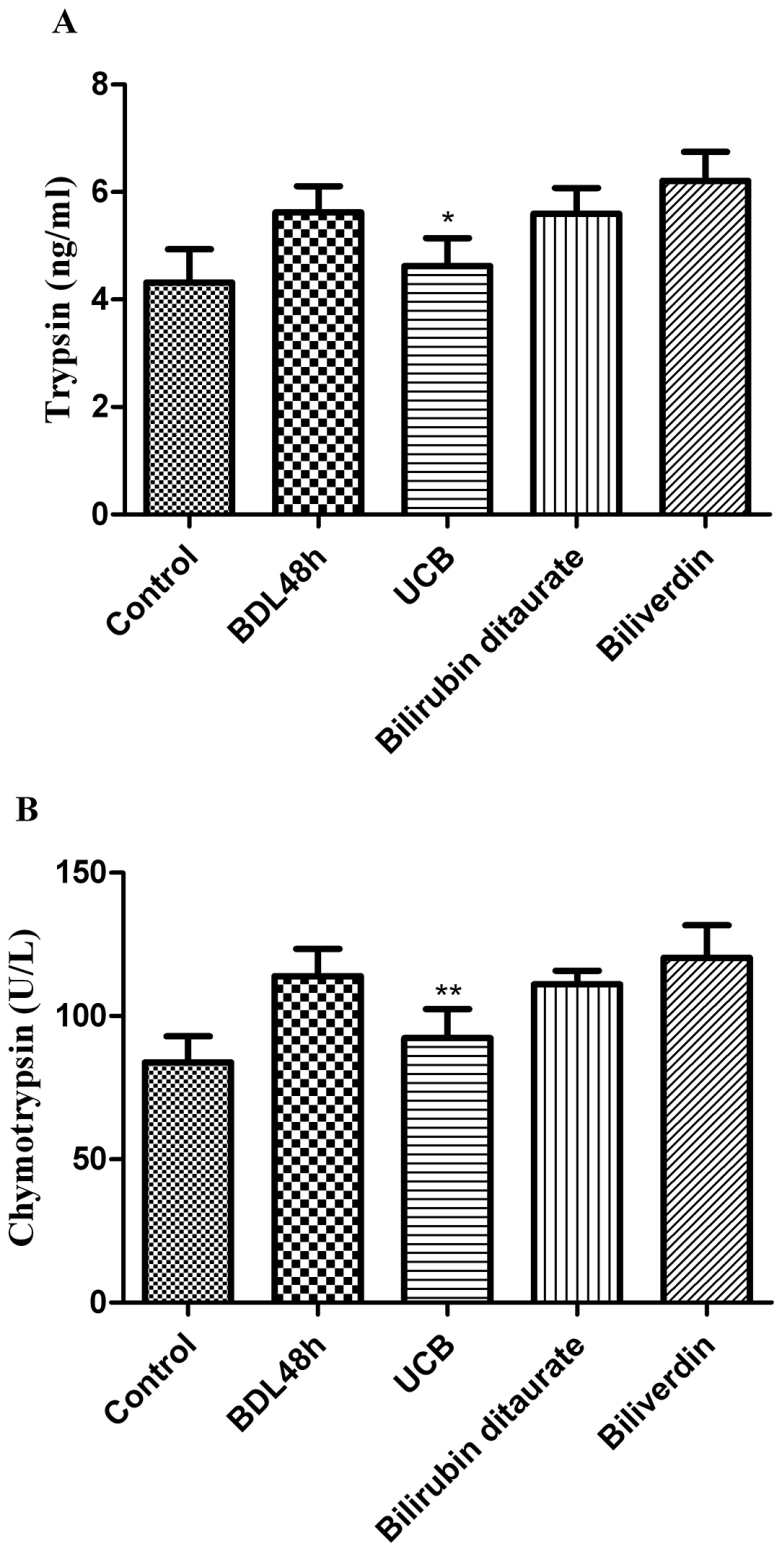
Effect of 0.1 mM bile pigments on trypsin, chymotrypsin levels. After ligation of bile duct, the animals were administrated with free bilirubin, bilirubin ditaurate, or biliverdin by intragastric gavage. The amounts of trypsin (A) and chymotrypsin (B) in middle small intestine were assayed by ELISA kit after 48 h. The data are expressed as mean ± SD from 8 independent animals, *p<0.05 **p<0.01 vs. respective control vs. respective BDL 48 h group.

As shown in Figure 4, 48 h after bilirubin ditaurate administration no changes in trypsin or chymotrypsin levels were observed in intestinal segment as compared to BDL 48 h group. The concentrations of trypsin, chymotrypsin in UCB administration group reduced by 17.8% (p<0.05) and 18.9% (p<0.01), respectively. On the contrary, these two digestive proteases levels were dramatically increased (increase of 10.4% and 5.6%, respectively) in biliverdin administration group. We can see that trypsin and chymotrypsin were inhibited by 0.1 mM UCB, but no significant changes were observed in bilirubin ditaurate administration group. In contrast, 0.1 mM biliverdin showed some stimulation on trypsin and chymotrypsin.

### Effect of 0.1 mM bile pigments on the compromised gut barrier function

Our prior results indicated that 0.1 mM of UCB possessed inhibitive property on trypsin and chymotrypsin. Next, we evaluate effect of 0.1 mM of different bile pigments on the permeability and integrity of intestinal mucosa and the histopathologic changes of intestinal tissues.

**Figure 5:**
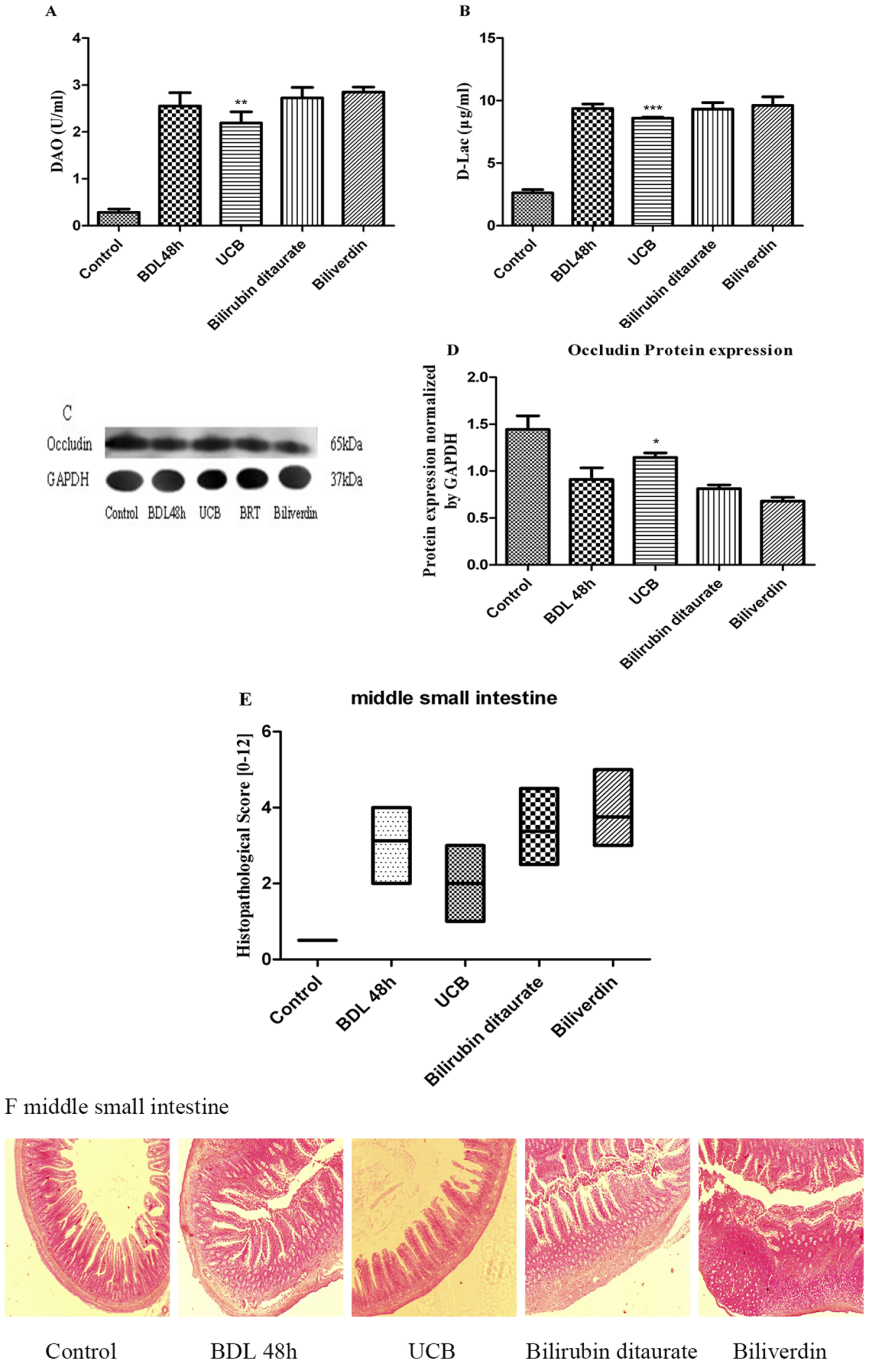
Effect of 0.1 mM bile pigments on the compromised gut barrier function. After ligation of bile duct, the animals were administrated with free bilirubin, bilirubin ditaurate, or biliverdin by intragastric gavage. The serum concentrations of DAO (A) and D-Lac (B) were assayed by spectrophotometric assay after 48 h. Protein expression of occludin (65 kDa) was analyzed by Western blot (C). Occludin bands were normalized by GAPDH and expressed as relative to BDL 48 h group (D). Intestinal slices were stained with HE staining and analyzed by inverted fluorescence microscope, middle small intestine were blindly assessed for the degree of histopathology (E), all photos were captured at ×40 magnification (F). Results are mean ± SD from at least three independent experiments. *p<0.05, **p<0.01 ***p<0.001 vs. respective BDL 48 h group.

After intragastric administration of UCB, bilirubin ditaurate and biliverdin, UCB administration group showed a significantly lower damage. DAO concentrations in UCB administration group reduced by 14.2%, p<0.01, while the serum concentrations of DAO in bilirubin ditaurate and biliverdin groups attained 7% and 11.7% increase, respectively (Figure 5A). In addition, the serum concentration of D-Lac in UCB administration group reduced by 8.2%, p<0.001. D-Lac levels in biliverdin administration group attained 3.6% increase, while no significant changes were observed in bilirubin ditaurate group (Figure 5B). As seen in Fig. 5C and Fig. 5D, the intestinal mucosa showed enhanced expression of occludin in UCB administration group only, as compared to BDL 48 h group (increase of 25.8%, p<0.05). At 48 h of BDL, the middle small intestine tissue pathology was significantly more severe compared to control group, with further aggravation and significantly more severely inflamed in 0.1 mM biliverdin treatment group (Figure 5E and 5F). The UCB treated group showed a remarkable decrease in intestinal histopathological score than BDL group. From this figure, we can see that 0.1 mM UCB but not bilirubin ditaurate or biliverdin displayed the protective effect on intestinal mucosa.

## Discussion

Here we demonstrate that bile may protect the gut from damage through inhibiting digestive proteases like trypsin and chymotrypsin by free bilirubin. We also show that UCB but not bilirubin ditaurate or biliverdin alleviates changes of histological and biochemical parameters related to gut barrier disruption.

The effect of digestive proteases on the intestinal barrier can switch from protective to disruptive. In addition, excessive protease release is a common feature of gut inflammatory disorders and plays a crucial role in generating gut barrier disruption by the digestion of the extracellular matrix and the alteration of the intestinal barrier homeostasis [24]. In this study, we found that the amounts of trypsin and chymotrypsin in middle small intestine were positively linearly correlated with DAO and D-Lac levels in serum. In addition, trypsin and chymotrypsin levels were negatively linearly correlated with the expression of occludin. Since these proteins are important indicators of intestinal permeability and integrity, bile duct ligation caused significant increases in gut trypsin and chymotrypsin along with damage of the mucosa.

Digestive proteases like trypsin and chymotrypsin could be inhibited by UCB, but not bilirubin ditaurate or biliverdin [12]. Bilirubin in the bile is mainly in the conjugated form, and conjugated bilirubin can be hydrolyzed to unconjugated bilirubin by β-glucuronidase, which exists in both eukaryotic cells and bacteria [15], [16]. Hydrolyzation of the conjugated biliary bilirubin would occur on the surface of the gut by β-glucuronidase released from the continuously sloughed off senescent epithelial cells of the mucosa, and the unconjugated bilirubin layer thus formed will exert an effective protection of the gut against the digestive damage by the luminal digestive proteases [25]. This may provide an explanation as to how the gut is protected against the damage by digestive proteases.

In the present study, anaesthetized adult male SD rats had their bile duct ligated. UCB stock solutions were further diluted with PBS and administered intragastrically at various concentrations. The dose-response study first revealed that UCB triggered a mild, but statistically significant, inhibition of DAO and D-Lac release, which attained minimum levels of 2.23 U/ml and 8.73 µg/ml at 0.1 mM UCB administration group, respectively. 0.1 mM of unconjugated bilirubin, this would be approximately 2.5-times that of the physiological output of biliary bilirubin under normal conditions [26].

The binding of unconjugated bilirubin to the catalytic site of alpha-chymotrypsin has been demonstrated, as indicated by circular dichroism (CD) displacement experiments performed with the competitive inhibitor acridine [13]. We showed that 0.1 mM UCB exerted a significant inhibition on trypsin and chymotrypsin, while no significant changes were observed in rats administered with 0.1 mM bilirubin ditaurate and biliverdin. Serine protease inhibitors include α_1_-antitrypsin, α_1_-antichymotrypsin, antithrombin III, elafin, protease-nexin and the universal protease inhibitor α_2_-macroglobulin [24]. We speculate that UCB may be a novel endogenous serine protease inhibitor, which remains to be determined. Our present study may provide a simple explanation for the energy-consuming conversion in some animals of the innocuous biliverdin to the water-insoluble, potentially toxic bilirubin [27], [28].

Bilirubin and biliverdin possess antioxidant and anti-inflammatory properties in experimental models [29], [30] and exogenous bilirubin and biliverdin supplements could be absorbed across the intestinal epithelium and potentially increase circulating concentrations of these antioxidant compounds [18]. In this study, 0.1 mM biliverdin showed some stimulation on trypsin and chymotrypsin and failed to exert the potential protective effect, which remains further investigating. In addition, we showed that UCB but not bilirubin ditaurate or biliverdin alleviated changes of histological and biochemical parameters related to gut barrier disruption.

In summary, this study shows the potential protective effect of UCB on intestinal mucosa through inhibiting digestive proteases. This may provide a new mechanism as to how the gut is protected against the damage by digestive proteases. Further research is needed to confirm our findings and to clarify the future mechanisms responsible for the protective effects of UCB on intestinal mucosa. Moreover, it is well documented that digestive proteases in the gut have played a critical role in the development of multiple organ dysfunction syndrome (MODS) under critically ill conditions and inflammatory bowel disease (IBD) that emerged and dramatically increased in last century. Our further research will focus on this field.
